# Atmospheric Pressure Plasma Polymerization Synthesis and Characterization of Polyaniline Films Doped with and without Iodine

**DOI:** 10.3390/ma10111272

**Published:** 2017-11-06

**Authors:** Choon-Sang Park, Eun Young Jung, Dong Ha Kim, Do Yeob Kim, Hyung-Kun Lee, Bhum Jae Shin, Dong Ho Lee, Heung-Sik Tae

**Affiliations:** 1 School of Electronics Engineering, College of IT Engineering, Kyungpook National University, Daegu 41566, Korea; purplepcs@ee.knu.ac.kr (C.-S.P.); eyjung@knu.ac.kr (E.Y.J.); ao9o9@ee.knu.ac.kr (D.H.K.); dhlee@ee.knu.ac.kr (D.H.L.); 2 ICT Materials and Components Research Laboratory, Electronics and Telecommunications Research Institute (ETRI), Daejeon 34129, Korea; nanodykim@etri.re.kr (D.Y.K.); hklee@etri.re.kr (H.-K.L.); 3 Department of Electronics Engineering, Sejong University, Seoul 05006, Korea; hahusbj@sejong.ac.kr

**Keywords:** atmospheric pressure plasma, plasma polymerization, iodine doping, nanofiber, polymerized aniline (PANI), X-ray Diffraction (XRD), X-ray photoelectron spectroscopy (XPS), time of flight secondary ion mass spectrometry (ToF-SIMS)

## Abstract

Although polymerized aniline (polyaniline, PANI) with and without iodine (I_2_) doping has already been extensively studied, little work has been done on the synthesis of PANI films using atmospheric pressure plasma (APP) deposition. Therefore, this study characterized pure and I_2_-doped PANI films synthesized using an advanced APP polymerization system. The I_2_ doping was conducted ex-situ and using an I_2_ chamber method following the APP deposition. The pure and I_2_-doped PANI films were structurally analyzed using field emission scanning electron microscope (FE-SEM), atomic force microscope (AFM), X-ray Diffraction (XRD), Fourier transform infrared spectroscopy (FT-IR), X-ray photoelectron spectroscopy (XPS), and time of flight secondary ion mass spectrometry (ToF-SIMS) studies. When increasing the I_2_ doping time, the plane and cross-sectional SEM images showed a decrease in the width and thickness of the PANI nanofibers, while the AFM results showed an increase in the roughness and grain size of the PANI films. Moreover, the FT-IR, XPS, and ToF-SIMS results showed an increase in the content of oxygen-containing functional groups and C=C double bonds, yet decrease in the C–N and C–H bonds when increasing the I_2_ doping time due to the reduction of hydrogen in the PANI films via the I_2_. To check the suitability of the conductive layer for polymer display applications, the resistance variations of the PANI films grown on the interdigitated electrode substrates were also examined according to the I_2_ doping time.

## 1. Introduction

Low-pressure plasma polymerization is a well-established and extensively studied area [1,2,3]. Use of such system, however, is costly and requires routine maintenance, thereby limiting its application to batch processes. Therefore, an attractive alternative is the use of atmospheric pressure plasma (APP) polymerization for the synthesis of novel thin film materials [4,5,6,7,8,9,10,11,12,13,14,15,16]. With various advantages, including a low-temperature dry process and low-cost routine maintenance, APP polymerization is a promising growth system for the deposition of novel plasma polymer thin films with functional features that are well-suited for a wide range of applications and substrates. Among plasma polymers, polyaniline (PANI) is a conducting polymer and promising candidate for use in organic electronics due to its low production cost, good electrical conductivity, semi-flexibility, and bio stability. In the case of display applications, it is already established that PANI can be obtained using chemical or low-pressure plasma polymerization methods [17]. The application of APP polymerization for fabricating PANI thin films, however, is not well developed.

The current authors recently reported on an advanced APP jets (APPJs) method for synthesizing new polymers [13,14,15,16] using intense plasma with a high plasma density in monomer fragmentation (or nucleation or active) and recombination (or passive) regions. The intense and broad plasma is produced using a guide tube and substrate on a polytetrafluoroethylene (PTFE) bluff body installed at the end of the jet, which minimizes any quenching from ambient air and increases the charged particles and density of the plasma in the fragmentation and recombination regions. In a dry and low temperature environment the proposed APP method can achieve a uniform and stable glow-like plasma discharge with a large area deposition and produce high-quality polymer nanoparticles and nanofibers with an easily controllable morphology, deposition rate, and deposition size, making them well-suited for a wide range of substrates [13,14,15,16]. Furthermore, the proposed APP polymerization technique does not require a vacuum atmosphere or special equipment. The uniform and intensified glow-like discharge of the novel APPJs technique was also shown to improve the fragmentation and recombination of the aniline monomer to produce pure plasma-polymerized aniline (pPANI) nanofibers including nanoparticles with high molecular weights and poly-crystalline characteristics [16]. The creation of novel polymer materials requires novel technological developments. In a previous study, the application of advanced APPJs without proton donor doping resulted in pure PANI in an insulated state. To create conductive plasma polymerized structures, charge carriers need to be introduced using I_2_ doping, which means that further analysis is required for the formation of novel pure and I_2_-doped PANI materials using the novel APPJs technique. In other research, Fourier transform infrared spectroscopy (FT-IR) and gas chromatography-mass spectrometry (GC-MS) were used to determine the chemical changes to pure PANI introduced by advanced APP. While FT-IR and GC-MS were useful in monitoring changes in the surface composition of pure PANI, there was no distinction between the nitrogen and carbon species arising from pure and I_2_-doped PANI. 

Accordingly, this study used atomic force microscopy (AFM), X-ray Diffraction (XRD), X-ray photoelectron spectroscopy (XPS), and time of flight secondary ion mass spectrometry (ToF-SIMS) to analyze the surface and grain size of pure and I_2_-doped PANI films and an FT-IR analysis to investigate the composition of conducting composites in the same films. In particular, XPS and ToF-SIMS are used as complementary surface analytical techniques for researching conducting polymer composites, and revealed interesting structural differences between the pure and I_2_-doped PANI films prepared in this study using advanced APPJs. Furthermore, the variation in resistance of the PANI films with and without I_2_ doping was also examined to check the suitability of the conductive layer for future display technologies.

## 2. Experimental Methods

### 2.1. Atmospheric Pressure Plasma Polymer Synthesis Method

High purity argon (Ar) gas (99.999%, Linde Korea, Pohang, Korea) was used as the plasma discharge gas, and its flow rate was 1700 standard cubic centimeters per minute (sccm). The liquid aniline monomer (Sigma-Aldrich Co., St. Louis, MO, USA, *M_w_* = 93 g·mol^−1^) was vaporized using a glass bubbler that supplied the Ar gas at a flow rate of 160 sccm. Sinusoidal power was applied to the powered electrode with a peak value of 8 kV and frequency of 26 kHz. The plasma polymerization method using an advanced APP source with a guide tube and bluff body was previously described in detail [13,16]. To measure the electrical resistance, the PANI films were synthesized on a substrate supplied with interdigitated Pt electrodes.

### 2.2. Iodine Doping Method

For ex-situ I_2_ doping, samples of the deposited PANI films were placed in a sealed glass container containing 2 g of solid I_2_ crystals (Sigma-Aldrich Co., St. Louis, MO, USA, 99.99%) for 30 s or 120 s. After doping, the color of the PANI films changed from light to dark brown.

### 2.3. Scanning Electron Microscopy

Plane and cross-sectional images of the plasma PANI films on the glass substrates were characterized using FE-SEM (Hitachi SU8220, Hitachi, Tokyo, Japan) with an accelerating voltage and current of 5 kV and 10 mA, respectively. A conductive platinum coating was used when imaging the samples to prevent any charging from the substrate.

### 2.4. Gel Permeation Chromatography

The gel permeation chromatography (GPC) was performed using an Alliance e2695 (Waters, Milford, MA, USA) GPC system. The polydispersity index (PDI) of the polymers was determined using Empower 2 software against polystyrene standards. Ultra-pure tetrahydrofuran (THF, 99.9% HPLC grade, Sigma-Aldrich Co., St. Louis, MO, USA) was used as an eluent, as well as a solvent to prepare the pure and I_2_-doped PANI solutions. To prevent any pressure fluctuation in the GPC system, the polymer solutions were filtered through a 25 mm polytetrafluoroethylene (PTFE) syringe filter prior to use.

### 2.5. Atomic Force Microscope

AFM (Park Systems NX20, Park Systems, Suwon, Korea) was used to monitor the surface roughness based on three-dimensional (3D) pure and I_2_-doped PANI surface images. The scan rate was set at 0.5 Hz and the scanning area was 20 μm × 20 μm.

### 2.6. X-Ray Diffraction

An XRD system (Bruker D6 Discover, Bruker, Billerica, MA, USA) was used to study the structural properties. The source of radiation was Cu-kα with wavelength λ = 1.5406 Å, voltage 40 kV, and current 40 mA. The scanning angle 2θ was varied in the range of 5–40°. The scan step size was set at 0.06° and the time per step was 5 s. 

### 2.7. Fourier Transform Infrared Spectroscopy

FT-IR was used to determine the chemical changes in the pure and I_2_-doped PANI. The FT-IR was taken using a Perkin-Elmer Frontier spectrometer between 650 cm^−1^ and 4000 cm^−1^. 

### 2.8. X-Ray Photoelectron Spectroscopy

The XPS was carried out using an ESCALAB 250Xi surface analysis system (Thermo Fisher Scientific, Waltham, MA, USA) with a monochromatic Al Kα X-ray source (*hv* = 1486.71 eV) operated at 15 kV and 20 mA. The pressure in the analyzing chamber was maintained at 10^−7^ Pa or lower, and the size of the analyzed area was 500 μm × 500 μm. The spectra were acquired using the angle between the direction of the emitted photoelectrons and the surface equal to 60°. The estimated analyzing depth of the XPS was 8 nm to 10 nm. The high-resolution spectra were acquired using the constant analyzer energy mode with 200 eV for the survey scan and 50 eV for the element scan. A C1s core level value of 285.8 eV was used for calibrating the energy scale.

### 2.9. Time of Flight Secondary Ion Mass Spectrometry

ToF-SIMS was used to investigate the surface composition of the polymers. The ToF-SIMS data were obtained using a ToF-SIMS V instrument (ION-TOF GmbH, Münster, Germany) equipped with a reflectron analyzer, bismuth primary-ion (Bi_3_^+^) source, and pulsed electron flood source for charge compensation. The pressure in the analysis chamber was maintained at less than 1 × 10^−9^ Torr. The negative-ion and positive-ion mass spectra were acquired from a 500 μm × 500 μm area using a Bi_3_^+^ (0.5 pA) primary-ion beam operating at 30 keV. The mass resolution was typically greater than 8000 at *m*/*z* = 29 Si. Secondary ions were detected in the negative-ion and positive-ion modes, and a full spectrum from 1 amu to 2000 amu was acquired.

## 3. Results and Discussion

**Figure 1:**
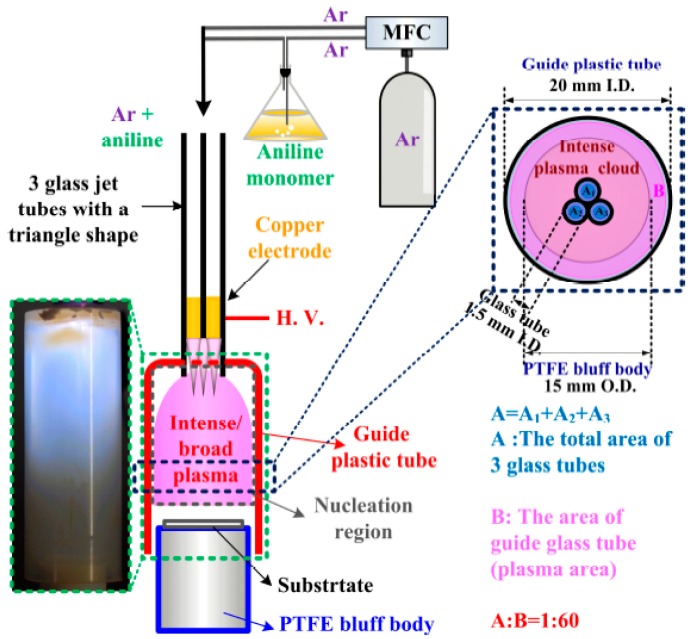
(**Left**) Schematic diagram of experimental set-up with plasma image produced in nucleation region of intense and broad plasma; and (**Right**) comparison of cross-sectional area of plasma, where A denotes restricted plasma area of three array jets and B denotes larger plasma area of intense and broad plasma region when using proposed advanced atmospheric pressure plasma (APP) polymerization system.

Figure 1 shows a schematic diagram of the experimental set-up with the plasma image produced in the nucleation region of the intense and broad plasma, and a comparison of the cross-sectional area of the plasma when using proposed advanced APP polymerization system. As shown in Figure 1 and previous research, the guide tube and bluff body systems are included in the proposed advanced APP polymerization system to minimize quenching from ambient air and increase the charged particles and plasma energy in the nucleation region [13,14,16]. Using copper tape, the three glass jet tubes were wrapped together to create a compact triangular shape, where each jet was in physical contact with the adjacent jets. A critical component of the advanced APP polymerization system is the guide tube, constituting the nucleation region where the aniline monomers are cracked by the Ar plasma. Moreover, the position of the bluff body with respect to the guide tube has a significant influence on the production of intense and broad plasma in the nucleation region. In the case of conventional APPJs without a guide tube or bluff body, plasma is only generated within the area of the three array jets due to the directional property of the streamer-like discharge. In the case of advanced APPJs (APP system) with a guide tube and bluff body, however, the plasma generated in the nucleation region can be transited from a narrow streamer-like discharge into a broad glow-like discharge. It is noted that, when generating intense and broad glow-like plasma in the guide tube, the plasma area was significantly increased about 60-fold, as shown in Figure 1. As a result, the advanced APPJs produced intense and broad glow-like plasma in the nucleation region during the plasma polymerization [13,16].

**Figure 2:**
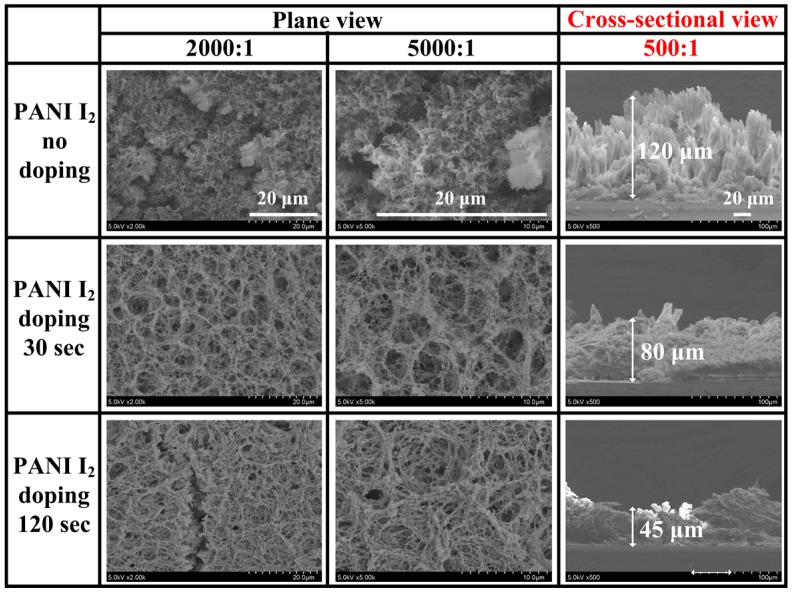
Plane (**left** and **middle**); and cross-sectional (**right**) scanning electron microscopy (SEM) images of polymerized aniline (PANI) films grown on glass substrates when using advanced APP jets (APPJs) device after 30 min deposition without iodine (I_2_), with 30 s I_2_ doping, and with 120 s I_2_ doping following deposition.

Figure 2 shows the changes in the plane and cross-sectional SEM images of the PANI films grown on glass substrates when using the advanced APPJs device after 30 min deposition without I_2_ doping (pure), with 30 s I_2_ doping, and with 120 s I_2_ doping following deposition. As shown in Figure 2, the pure PANI films consisted of dense nanoparticles and nanofibers with irregularly cross-linked and porous networks with an excellent PDI of about 1.09 according to GPC, whereas the I_2_-doped PANI films showed irregularly low cross-linked and highly porous networks with a PDI of 1.11. In particular, after I_2_ doping, the plane SEM images showed narrower PANI nanofibers, while the cross-sectional SEM images showed thinner PANI nanofibers and films when increasing the I_2_ doping time. After 120 s of doping, some nanofibers snapped between adjacent nanofibers.

**Figure 3:**
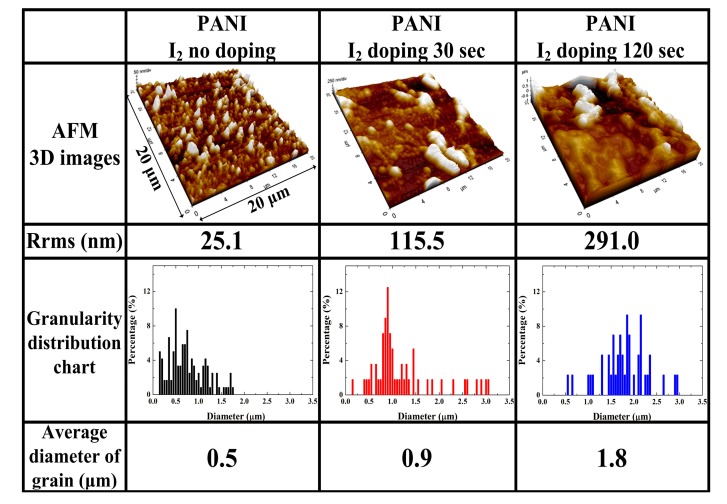
Three-dimensional (3D) atomic force microscope (AFM) images of roughness (R_rms_) of PANI film surfaces grown on glass substrates and granularity cumulation distribution charts with average grain diameter obtained from AFM images when using advanced APPJs device after 30 min deposition without I_2_ doping, with 30 s I_2_ doping, and with 120 s I_2_ doping following deposition.

Figure 3 shows the changes in the 3D AFM images according to the roughness (R_rms_) of the PANI film surface grown on a glass substrate and granularity cumulation distribution charts of the average grain diameter obtained from the AFM images when using the advanced APPJs device after 30 min deposition without I_2_ doping, with 30 s I_2_ doping, and with 120 s I_2_ doping following deposition. The pure PANI surface showed small-size protrusions, and the surface R_rms_ was 25.1 nm, whereas after I_2_ doping, lots of large protrusions appeared on the PANI surface, and the R_rms_ increased to 115.5 nm and 291.0 nm, respectively. The surface became rougher when increasing the doping time. In addition, as shown in the granularity cumulation distribution charts in Figure 3, the diameter range for the pure PANI sample was between 0.15 μm and 1.75 μm. After 120 s of I_2_ doping, however, the diameter range became much wider, between 0.55 μm and 2.95 μm, due to particle aggregation, thereby increasing the grain size [18,19,20]. As the grain sizes determine the number of grain boundaries, the increase in the average grain diameter from 0.5 μm to 1.8 μm means that increasing the I_2_ doping time increases the grain size and simultaneously decreases the number of grain boundaries. The 3D AFM images also indicated that the PANI film surfaces became rougher when increasing the I_2_ doping; this increased roughness was mainly due to particle aggregation and a higher porosity due to disconnected networks between adjacent nanofibers.

**Figure 4:**
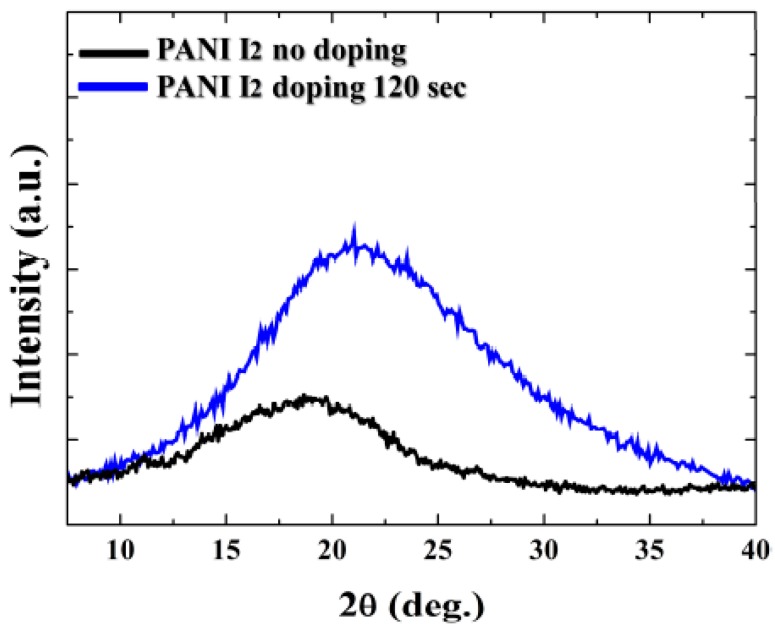
X-ray diffraction (XRD) patterns of PANI film surfaces grown on Si substrates using advanced APPJs device after 60 min deposition without I_2_ doping and with 120 s I_2_ doping following deposition.

Figure 4 shows the changes in the XRD patterns of the PANI films surface grown on Si substrates when using the advanced APPJs device after 60 min deposition without I_2_ doping and with 120 s I_2_ doping following deposition. The PANI XRD patterns showed that the pure PANI was essentially amorphous in terms of a film [21,22]. There was only one broad diffraction peak at around 19.0°. After 120 s of I_2_ doping, however, a broad diffraction peak appeared at around 20.9°, and the intensity of the peak at around 20.9° increased. 

**Figure 5:**
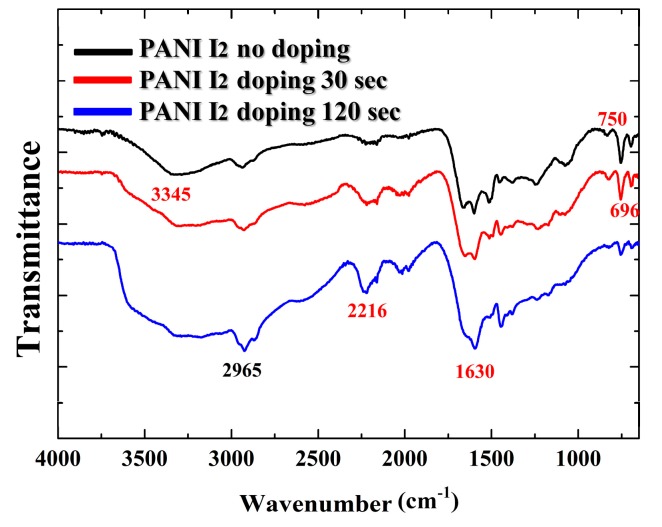
Fourier transform infrared spectroscopy (FT-IR) spectra of PANI films grown on glass substrates using advanced APPJs device after 60 min deposition without I_2_ doping, with 30 s I_2_ doping, and with 120 s I_2_ doping following deposition.

Figure 5 shows the changes in the FT-IR spectra of the PANI films grown on glass substrates when using the advanced APPJs device after 60 min deposition without I_2_ doping, with 30 s I_2_ doping, and with 120 s I_2_ doping following deposition. The first broad band located at 3345 cm^−1^ corresponds to N–H bonds with hydrogen bonded secondary amino groups. A peak belonging to aliphatic C–H groups was also located at 2965 cm^−1^ [23,24,25]. When increasing the I_2_ doping time, the groups with multiple bonds C≡C in the peak at 2216 cm^−1^ were increased along with the C=C double bonds in the sharp peak at 1630 cm^−1^ [26,27,28,29], whereas the C–N bonds of the diphenyl amine group were decreased in the sharp peaks at 750 cm^−1^ and 696 cm^−1^. The presence of C=C (1630 cm^−1^) bonds can increase the removal of hydrogen from consecutive carbon atoms and the subsequent rearrangement of radicals. Therefore, as shown in Figure 5, the FT-IR results showed that the C≡C and C=C double bonds were increased and the C–N bonds decreased when increasing the I_2_ doping time, presumably due to the reduction of hydrogen in the PANI films via the iodine [26], which probably reacted with residual radicals in the PANI films during the ex-situ doping [30,31]. Iodine radicals can also be formed by the hemolytic dissociation of iodine [30]. As iodine radicals can extract hydrogen atoms from the PANI structure, this could change the bonding characteristics of plasma polymer films [30,31,32].

**Figure 6:**
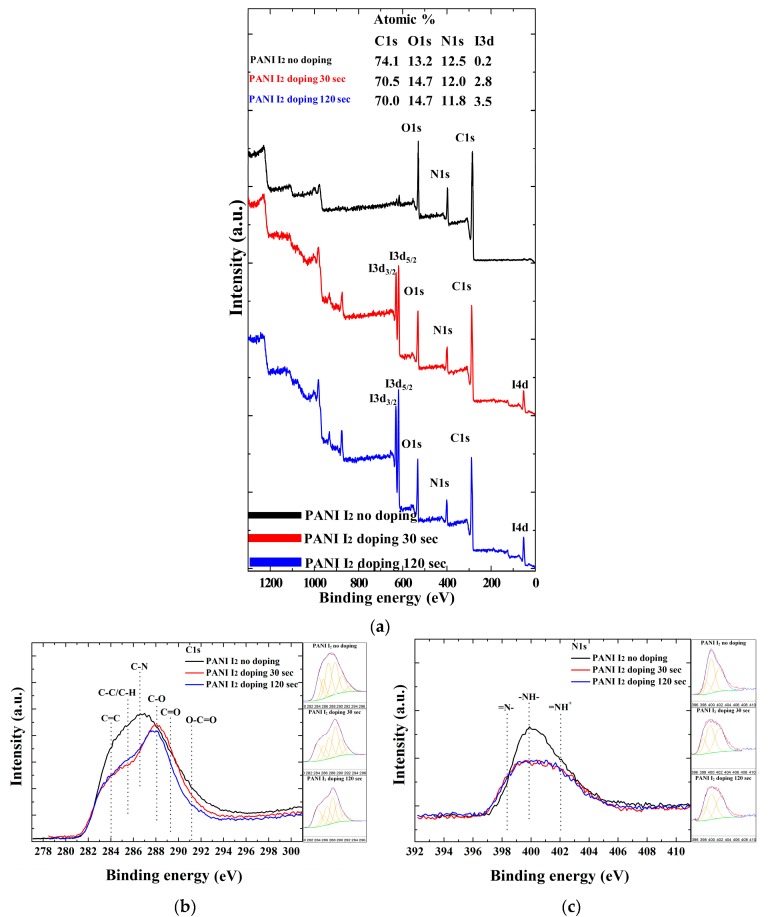

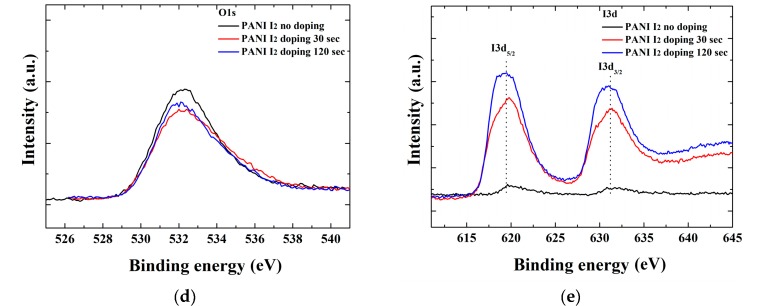
Differences in PANI film surfaces grown on glass substrates when using advanced APPJs device after 60 min deposition without I_2_ doping, with 30 s I_2_ doping, and with 120 s I_2_ doping following deposition: (**a**) X-ray photoelectron spectroscopy (XPS) survey spectra; and detailed (**b**) C 1s (high-resolution); (**c**) N 1s (high-resolution); (**d**) O 1s; and (**e**) I 3d spectra. Insets in (**a**) represent atom percent in PANI film.

Figure 6 shows the XPS spectra and elemental composition percent (Figure 6a, insets) of the atomic distribution in the PANI films grown on glass substrates when using the advanced APPJs device after 60 min deposition without I_2_ doping, with 30 s I_2_ doping, and with 120 s I_2_ doping following deposition. The element content was calculated based on the ratio of the corresponding integral area of the peaks. The XPS survey spectra in Figure 6a show signals corresponding to C 1s (285.5 eV), N 1s (399.8 eV), O 1s (532.1 eV), I 3d_3/2_ (631.1 eV), I 3d_5/2_ (619.7 eV), and I 4d (53.0 eV) electronic orbitals [33,34,35]. These results suggest the presence of C, N, I, and O atoms in the pure and I_2_-doped PANI films. While the C, N, and I atoms belong to the aniline and iodine structures, the O atoms are likely to originate from the oxidation of particles from ambient air during the APP polymerization and I_2_ doping. For a further analysis of the types and ratios of the surface functional groups in the pure and I_2_-doped PANI, the curve fitting of the high-resolution C 1s and N 1s peaks was analyzed using the XPS peaks. As shown in Figure 6b, the C 1s peaks for the pure and I_2_-doped PANI could be divided into six component peaks, indicating the following six carbon-containing groups; C=C (284.0 eV), C–C/C–H (285.6 eV), C–N (286.5 eV), C–O (288.1 eV), C=O (289.4 eV), and O–C=O (291.1 eV) [5,26,27]. Meanwhile, the N 1s spectra under different conditions are shown in Figure 6c. In this case, the N 1s peaks for the pure and I_2_-doped PANI could be divided into three component peaks, indicating the following three nitrogen-containing groups; =N– (398.3 eV), –NH– (399.8 eV), and =NH^+^ (402.1 eV) [27,36,37]. The assignments of the fitted components and compositions of the C 1s and N 1s levels are summarized in Table 1 and Table 2, respectively. As shown in Figure 6a, in the case of pure PANI, the C 1s, N 1s, O 1s, and related I 3d peaks had an atom percent of 74.1%, 12.5%, 13.2%, and 0.2%, respectively. In the case of 120 s of I_2_ doping, however, the C 1s, N 1s, O 1s, and related I 3d peaks had an atom percent of 70.0%, 11.8%, 14.7%, and 3.5%, respectively, which meant that, when increasing the I_2_ doping time, the C 1s and N 1s decreased, but the O 1s and I 3d increased. As shown in Figure 6b and Table 1, when the I_2_ doping time was 30 s, the C=C content increased from 16.84% to 19.25%. When the I_2_ doping time reached 120 s, the C=C content increased from 16.84% to 22.47%. Furthermore, the content of oxygen-containing groups, such as C=O, O–C=O, increased when increasing the I_2_ doping time. When the I_2_ doping time was 120 s, however, the content of C–C/C–H and C–N decreased from 11.54% and 26.00% to 10.86% and 19.48%, respectively. As shown in Figure 6c and Table 2, when the I_2_ doping time was 120 s, the content of –NH– and NH_2_^+^ also decreased from 44.04% and 42.12% to 42.84% and 39.31%, respectively. As a result, when increasing the I_2_ doping time, the oxygen-containing functional groups and C=C double bonds all increased, while the C–N, C–H, –NH–, and =NH^+^ bonds all decreased. The primary carbon-containing bonds were decomposed by the APP, which helped to generate oxidation, carboxyl, and double bonds of carbon to oxygen under ambient air, thereby increasing the content of oxygen-containing functional groups and C=C double bonds. Meanwhile, since iodine could easily absorb hydrogen from materials, this reduced the C–H, C–N, –NH–, and =NH^+^ bonds. In addition, the C=C bonds were likely to be increased with the removal of hydrogen of consecutive carbon atoms [26]. 

**Figure 7:**
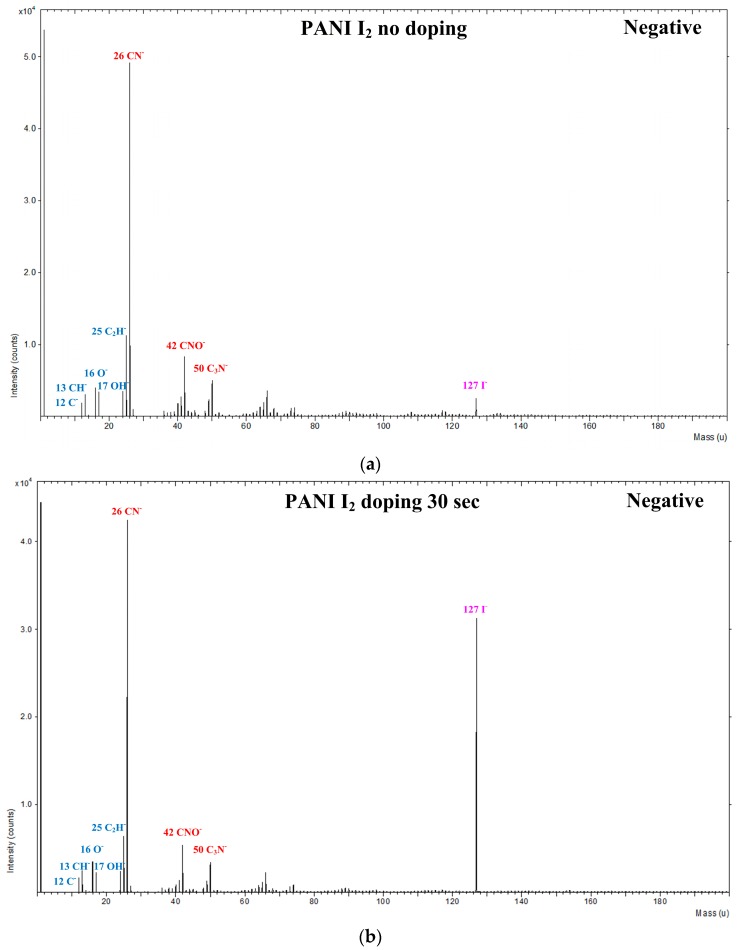

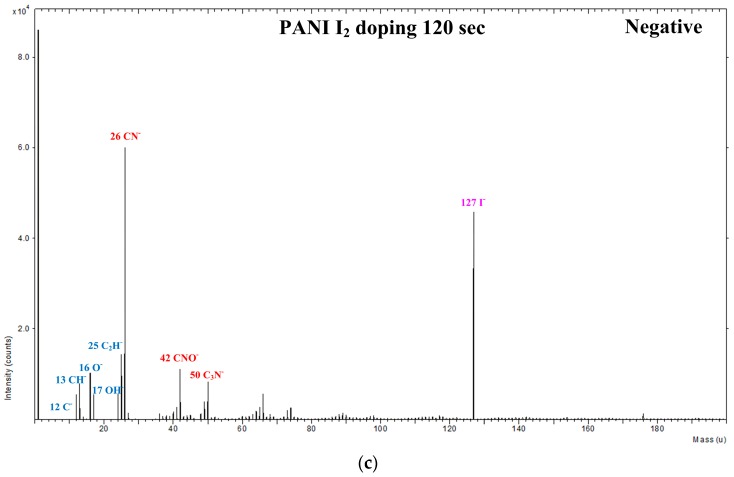
Negative-ion time of flight secondary ion mass spectrometry (ToF-SIMS) spectra (0–200 amu) of PANI film surfaces grown on glass substrates when using advanced APPJs device after 60 min deposition: (**a**) without I_2_ doping; (**b**) with 30 s I_2_ doping; and (**c**) with 120 s I_2_ doping following deposition.

To determine the specific structures with and without I_2_ doping after the APP polymerization process, the PANI films were characterized in both the positive and negative ion modes using ToF-SIMS. Figure 7 shows the negatively charged ions with wide range (0–200 amu) static mass spectra when using ToF-SIMS for the PANI films grown on glass substrates when using the advanced APPJs device after 60 min deposition without I_2_ doping, with 30 s I_2_ doping, and with 120 s I_2_ doping following deposition. The spectra were complex and showed distinct differences, attesting to substantial compositional differences between the pure and I_2_-doped PANI films. Two ion peaks were detected at *m*/*z* = 16 and 17 amu, corresponding to O^−^ and OH^−^, respectively. In addition, PANI ions were detected at *m*/*z* = 26, 42, and 50 amu and attributed to CN^−^, CNO^−^, and C_3_N^−^, respectively, which were characteristic of PANI fragments. The assignment of the selected peaks of PANI detected in the negative mode is shown in Table 3.

**Figure 8:**
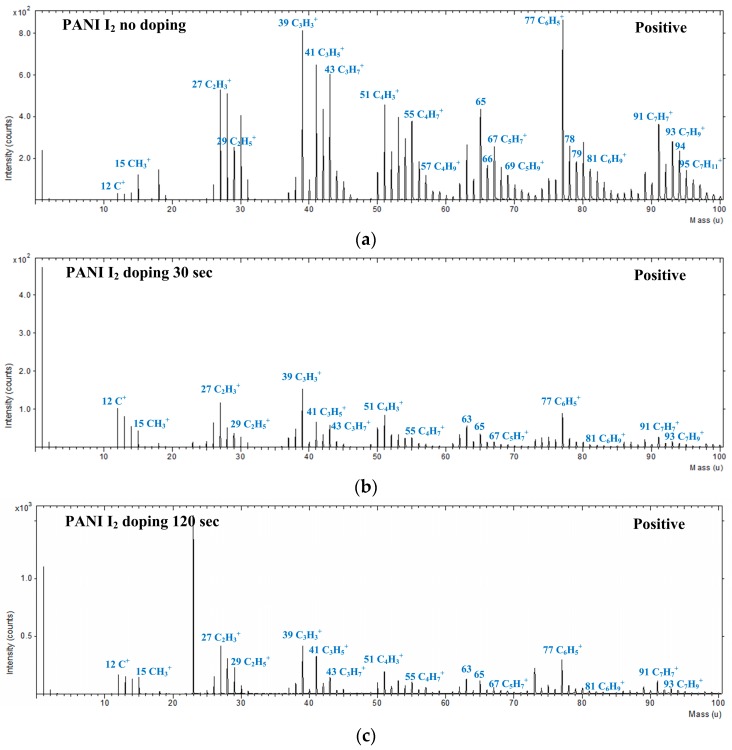
Positive-ion ToF-SIMS narrow range spectra (0–100 amu) of PANI film surfaces grown on glass substrates using advanced APPJs device after 60 min deposition: (**a**) without I_2_ doping; (**b**) with 30 s I_2_ doping; and (**c**) with 120 s I_2_ doping following deposition.

Figure 8 shows the positively charged ions with narrow range (0–100 amu) static mass spectra for the PANI films grown on a glass substrate when using the advanced APPJs device after 60 min deposition without I_2_ doping, with 30 s I_2_ doping, and with 120 s I_2_ doping following deposition. For the PANI features, several characteristic peaks from the polymer chain were detected. Table 4 lists the selected peak assignments for PANI detected in the positive ion mode, representing a series of hydrocarbon fragments arising from PANI, such as C_2_H_5_^+^, C_3_H_5_^+^, C_3_H_7_^+^, and C_4_H_7_^+^. These clusters of peaks at 39, 41, 43, 55, 57, 67, 69, 81, and 95 amu are typical aliphatic hydrocarbon fragments of the form C_n_H_2n__−__3_, C_n_H_2n__−__1_, and C_n_H_2n+1_, arising from the PANI chain.

**Figure 9:**
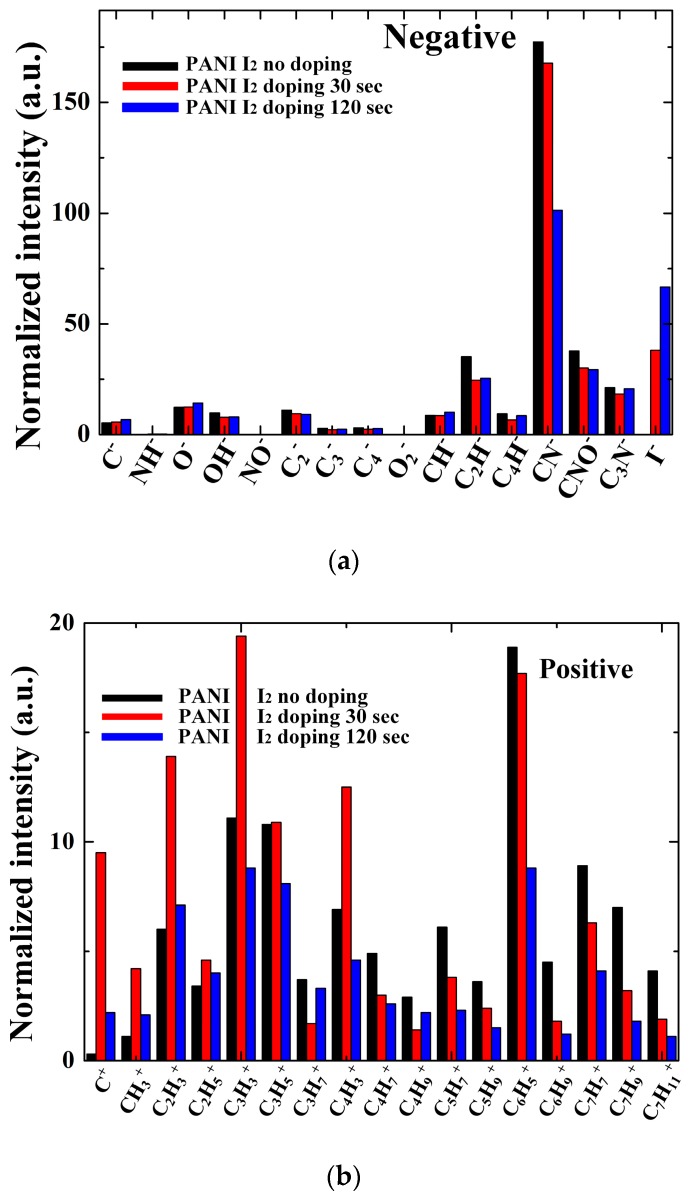
Comparison of normalized intensities of: (**a**) negative-ion ToF-SIMS; and (**b**) positive-ion ToF-SIMS on surface of PANI films when using advanced APPJs device without I_2_ doping, with 30 s I_2_ doping, and with 120 s I_2_ doping following deposition.

Figure 9 shows the changes in the normalized intensities of the negative-ion and positive-ion ToF-SIMS on the surface of the PANI films when using the advanced APPJs device after 60 min deposition without I_2_ doping, with 30 s I_2_ doping, and with 120 s I_2_ doping following deposition. As shown in Figure 9a, increasing the I_2_ doping time produced a significant decrease in CN^−^, CNO^−^, and C_3_N^−^, and slight decrease in OH^−^, C_2_H^−^, and C_4_H^−^. In Figure 9b, all the aliphatic hydrocarbon fragment spectra, including C_3_H_3_^+^, C_3_H_5_^+^, C_3_H_7_^+^, C_4_H_7_^+^, C_4_H_9_^+^, C_5_H_7_^+^, C_5_H_9_^+^, C_6_H_9_^+^, and C_7_H_11_^+^, decreased when increasing the I_2_ doping time to 120 s. As a result, since both the nitrogen-containing groups and the hydrogen-containing groups were decreased, this reduced the C–H and C–N bonds. This result agreed well with the FT-IR and XPS analyses.

**Figure 10:**
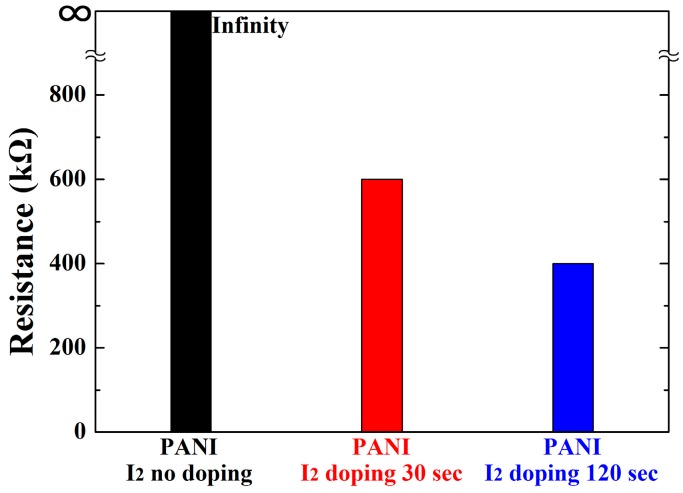
Changes in resistance of PANI films grown on substrates of interdigitated electrodes when using advanced APPJs device after 60 min deposition with no I_2_ doping, with 30 s I_2_ doping, and with 120 s I_2_ doping following deposition.

Figure 10 shows the changes in the resistance (*R*) of the PANI films on the substrates of interdigitated electrodes when using the advanced APPJs device after 60 min deposition without I_2_ doping, with 30 s I_2_ doping, and with 120 s I_2_ doping following deposition. The width and gap of the interdigitated electrodes were about 330 and 230 μm, respectively. The purpose of doping with iodine was to introduce charge carriers to the PANI structure to enhance the electrical conductive characteristics. The samples were doped by placing them in a sealed glass container containing solid I_2_ crystals (2 g) for a certain time period [30,31,32]. The resistance of the pure PANI film was over the measurement limit of 1.0 × 10^8^ Ω. When increasing the doping time, the corresponding resistance decreased mainly due to the increased charge carriers and grain size. As shown in Figure 10, when the I_2_ doping time was 30 s, the corresponding resistance was remarkably decreased from infinity to 6.0 × 10^5^ Ω. When the I_2_ doping time reached 120 s, the corresponding resistance was more slightly decreased from 6.0 × 10^5^ Ω to 4.0 × 10^5^ Ω. The corresponding resistance was saturated at about 1.0 × 10^3^ Ω after 20 min. In most polymer materials, the presence of water in or on the polymer structure changes the resistance and conductivity [15,16,17,18]. In polymer display applications, however, the I_2_-doped PANI film would be encapsulated by sealing materials to prevent access by moisture and oxygen, thereby protecting the resistance and conductivity [38,39,40]. For polymer display applications, a future detailed parametric study will measure the electric conductivity characteristics of plasma polymers created using the proposed advanced APPJs device.

## 4. Conclusions

Pure and I_2_-doped PANI films were prepared using an advanced APP polymerization system to study the formation of thin films for potential application. The I_2_ doping was conducted ex-situ and using an I_2_ chamber method after APP polymerization deposition. FE-SEM and AFM studies revealed that the thickness of the PANI films decreased while the roughness increased when increasing the I_2_ doping time; this increased roughness was mainly due to a high porosity from disconnected networks between adjacent nanofibers. FT-IR, XPS, and ToF-SIMS revealed that increasing the I_2_ doping time increased the content of oxygen-containing functional groups and C=C double bonds, yet decreased the C–N, C–H, –NH–, and =NH^+^ bonds due to the reduction of hydrogen in the PANI films via the I_2_ doping. For polymer display applications, the resistance of PANI films can be easily controlled by varying the I_2_ doping time. It is also anticipated that new PANI films grown at room temperature (30 °C) using the proposed advanced APP system can offer versatile advantages as electrodes for future displays, flexible plasma thrusters, and polymer gas sensors.

## Figures and Tables

**Table 1:** Peak assignment (BE, eV) and envelope composition (%, total = 100) of various C 1s core level spectra of polymerized aniline (PANI) observed in X-ray photoelectron spectroscopy (XPS) spectra in Figure 6b.

Sample	C 1s Peaks Assignment and Envelope Composition					
	284.0 C=C	285.6 C–C/C–H	286.5 C–N	288.1 C–O	289.4 C=O	291.1 O–C=O
PANI I_2_ no Doping	16.84	11.54	26.00	26.79	14.73	4.10
PANI I_2_ Doping 30 s	19.25	12.77	20.48	27.27	13.80	6.43
PANI I_2_ Doping 120 s	22.47	10.86	19.48	22.46	17.97	6.76

**Table 2:** Peak assignment (BE, eV) and envelope composition (%, total = 100) of various N 1s core level spectra of PANI observed in XPS spectra in Figure 6c.

Sample	N 1s Peaks Assignment and Envelope Composition		
	398.3 =N–	399.8 –NH–	402.1 =NH^+^
PANI I_2_ no Doping	13.84	44.04	42.12
PANI I_2_ Doping 30 s	17.02	42.17	40.81
PANI I_2_ Doping 120 s	17.85	42.84	39.31

**Table 3:** Selected peaks and their assignments observed in negative-ion time of flight secondary ion mass spectrometry (ToF-SIMS) spectra of PANI.

Negative Ion Mass Spectrum	Possible Ion Fragment/Possible Structure
*m/z*	
12	C^-^
13	CH^−^
15	NH^−^
16	O^−^
17	OH^−^
24	C_2_^−^
25	C_2_H^−^
26	CN^−^
30	CNO^−^
32	O_2_^−^
36	C_3_^−^
37	C_3_H^−^
42	CNO^−^
48	C_4_^−^
49	C_4_H^−^
50	C_3_N^−^
127	I^−^

**Table 4:** Selected peaks and their assignments observed in positive-ion ToF-SIMS spectra of PANI.

Positive Ion Mass Spectrum	Possible Ion Fragment/Possible Structure
*m/z*	
12	C^+^
15	CH_3_^+^
27	C_2_H_3_^+^/CH_2_–CH^+^
29	C_2_H_5_^+^/CH_3_–CH_2_^+^
39	C_3_H_3_^+^/CH_2_–C–CH^+^
41	C_3_H_5_^+^/CH_2_–CH–CH_2_^+^
43	C_3_H_7_^+^/CH_3_–CH_2_–CH_2_^+^
51	C_4_H_3_^+^/CH_2_–C–C–CH^+^
55	C_4_H_7_^+^/CH_2_–CH–CH_2_–CH_2_^+^
57	C_4_H_9_^+^ or C_3_H_7_N^+^
67	C_5_H_7_^+^
69	C_5_H_9_^+^
77	C_6_H_5_^+^
81	C_6_H_9_^+^
91	C_7_H_7_^+^
93	C_7_H_9_^+^
95	C_7_H_11_^+^
